# Revising model for end-stage liver disease from calendar-time cross-sections with correction for selection bias

**DOI:** 10.1186/s12874-024-02176-8

**Published:** 2024-02-28

**Authors:** H. C. de Ferrante, M. van Rosmalen, B. M. L. Smeulders, S. Vogelaar, F. C. R. Spieksma

**Affiliations:** 1 Department of Mathematics and Computer Science, Eindhoven University of Technology, 5600MB Eindhoven, PO Box 513, Netherlands; 2 Eurotransplant International Foundation, Leiden, the Netherlands

**Keywords:** Eurotransplant, Urgency-based liver allocation, Dependent censoring, Landmarking, Inverse probability censoring weighting, Chronic liver cirrhosis, Liver allocation, Partly conditional models, Informative censoring

## Abstract

**Background:**

Eurotransplant liver transplant candidates are prioritized by Model for End-stage Liver Disease (MELD), a 90-day waitlist survival risk score based on the INR, creatinine and bilirubin. Several studies revised the original MELD score, UNOS-MELD, with transplant candidate data by modelling 90-day waitlist mortality from waitlist registration, censoring patients at delisting or transplantation. This approach ignores biomarkers reported after registration, and ignores informative censoring by transplantation and delisting.

**Methods:**

We study how MELD revision is affected by revision from calendar-time cross-sections and correction for informative censoring with inverse probability censoring weighting (IPCW). For this, we revised UNOS-MELD on patients with chronic liver cirrhosis on the Eurotransplant waitlist between 2007 and 2019 (*n *= 13,274) with Cox models with as endpoints 90-day survival (a) from registration and (b) from weekly drawn calendar-time cross-sections. We refer to the revised score from cross-section with IPCW as *DynReMELD*, and compare *DynReMELD* to UNOS-MELD and ReMELD, a prior revision of UNOS-MELD for Eurotransplant, in geographical validation.

**Results:**

Revising MELD from calendar-time cross-sections leads to significantly different MELD coefficients. IPCW increases estimates of absolute 90-day waitlist mortality risks by approximately 10 percentage points. DynReMELD has improved discrimination over UNOS-MELD (delta c-index: 0.0040, *p* < 0.001) and ReMELD (delta c-index: 0.0015, *p* < 0.01), with differences comparable in magnitude to the addition of an extra biomarker to MELD (delta c-index: ± 0.0030).

**Conclusion:**

Correcting for selection bias by transplantation/delisting does not improve discrimination of revised MELD scores, but substantially increases estimated absolute 90-day mortality risks. Revision from cross-section uses waitlist data more efficiently, and improves discrimination compared to revision of MELD exclusively based on information available at listing.

**Supplementary Information:**

The online version contains supplementary material available at 10.1186/s12874-024-02176-8.

## Introduction

Eurotransplant prioritizes liver transplant candidates by the Model for End-stage Liver Disease (MELD) score, a disease severity scoring system based on serum bilirubin, serum creatinine, and the INR (see [1] for a description of ET liver allocation). MELD was first used by UNOS for liver allocation after external validations showed the originally proposed MELD score, UNOS-MELD, predicted 90-day waitlist mortality well for cirrhotic patients [2, 3]. Since then various limitations of UNOS-MELD have been described, including that

1. UNOS-MELD was not developed for prediction of waitlist mortality of liver transplant candidates [4],
2. UNOS-MELD overemphasizes renal dysfunction [5],
3. Caps imposed on UNOS-MELD biomarkers were based on medical intuition [6],
4. UNOS-MELD is poorly calibrated for certain subgroups, notably hyponatremic patients [7].

Such limitations have motivated several studies to revise MELD, either by updating the equation’s coefficients with liver waitlist candidate registry data (e.g., [5, 6]), or by expanding the scoring system with new biomarkers (e.g., MELD-Na [7] and MELD 3.0 [8]). Recently, UNOS-MELD was revised specifically for Eurotransplant, leading to the ReMELD score [9].

MELD revision typically proceeds by modelling waitlist mortality up to 90 days after waitlist registration based on biomarkers reported at registration (e.g., [6–9]). This *“from registration”* poorly aligns with clinical use of MELD, as liver transplant candidates are prioritized by the last reported MELD score and not MELD at registration. Moreover, revising MELD *“from registration”* ignores waitlist deaths occurring more than 90 days after listing (two thirds of total waitlist deaths in Eurotransplant), and ignores patient conditions reported after registration, thereby inefficiently uses available waitlist registry data.

Previously such waste of statistical information was avoided by adjusting for MELD biomarkers as time-varying covariates (e.g., [5, 10]). However, MELD biomarkers also increase intrinsically as part of the death process [11], such that adjustment for MELD biomarkers as time-varying covariates leads to issues of reverse causality. This reverse causality problem is aggravated by the fact that sicker patients are required to update their MELD scores more frequently and that MELD scores can be updated voluntarily at any time.

To avoid these issues we propose to revise MELD *“from cross-section”* based on methodology proposed by Gong & Schaubel [12]. With this approach MELD is revised by modelling the remaining time-until-death from pre-specified calendar-time cross-sections rather than from registration. Biomarker measurements collected after listing and deaths recorded more than 90 days after listing thus inform MELD revision. To avoid issues of reverse causality adjustment at each cross-section is for historic biomarker information. Biomarkers recorded after the cross-section date do affect survival and transplantation/delisting rates, making transplantation/delisting informative censoring mechanisms. Prior revisions of MELD censor patients at transplantation/delisting, essentially ignoring the bias due to informative censoring. We study how MELD revision is affected by correction for dependent censoring with inverse probability censoring weighting, also proposed by [12].

## Material and methods

The Ethical Review Board of Eindhoven University of Technology approved the study, and waivered informed consent.

### Study population & data

Adult patients with any active waitlist status on the Eurotransplant waiting list between 16–12-2006 and 31–12-2019 were retrieved from Eurotransplant. Only patients with chronic liver cirrhosis were included for the study, i.e. the group on which MELD-UNOS was originally validated. Patients with other diagnoses, priority due to exception points, and patients waiting for a re-transplantation or combined transplantation (except kidney) were excluded. Patients with impossible values for MELD biomarkers (e.g., all zeroes for INR, creatinine, and bilirubin) were also excluded.

Reporting of MELD biomarkers (creatinine, INR, bilirubin, dialysis) is mandatory at Eurotransplant liver waitlist registration. Reported MELD scores expire within at most 1 year, and more rapidly for sicker patients (within 7 days for MELD scores greater than 25 [13]). Failure to update the MELD score results in the lowest possible MELD score of 6 being used for allocation. Updates to MELD scores are therefore available for most transplant candidates. Candidates temporarily unavailable for transplantation can be set to non-transplantable (NT).

### MELD scores, UNOS-MELD and ReMELD

The MELD scoring system calculates the score based on serum creatinine, serum total bilirubin and the INR as

$${\text{intercept}} + {\text{coef}}_{\text{crea}}\log\!\left({\text{crea}}\right)+{{\text{coef}}}_{\text{bili}}\log\!\left({\text{bili}}\right)+{{\text{coef}}}_{\text{INR}}\log\!\left({\text{INR}}\right),$$

with serum creatinine and bilirubin measured in mg/dL. A specific MELD score proposes values for the intercept and coefficients, bounds for the values of MELD biomarkers, and how to set creatinine for patients on dialysis. Eurotransplant currently uses UNOS-MELD for allocation, i.e.

$$6.43 + 9.57\log\!\left({\text{crea}}\right)+3.78\log\!\left({\text{bili}}\right)+11.20\log\!\left({\text{INR}}\right),$$

with creatinine capped at 4.0 mg/dL, a lower limit of 1.0 imposed on all biomarkers, and creatinine set to 4.0 for patients on biweekly dialysis.

Various revisions of MELD have been proposed (e.g., [6, 9, 11]). One alternative developed specifically for Eurotransplant is ReMELD [9], which calculates the score as

$$8.422 + 7.728 \log\!\left({\text{crea}}\right)+3.446\log\!\left({\text{bili}}\right)+10.597\log\!\left({\text{INR}}\right),$$

With bilirubin bounded to 0.3–27 mg/dL, INR bounded to 0.1–2.6, creatinine bounded to 0.7–2.5 mg/dL and set to 2.5 mg/dL if the patient is on biweekly dialysis.

### Revision *“from registration” vs. “revision from cross-section”*

**Fig. 1:**
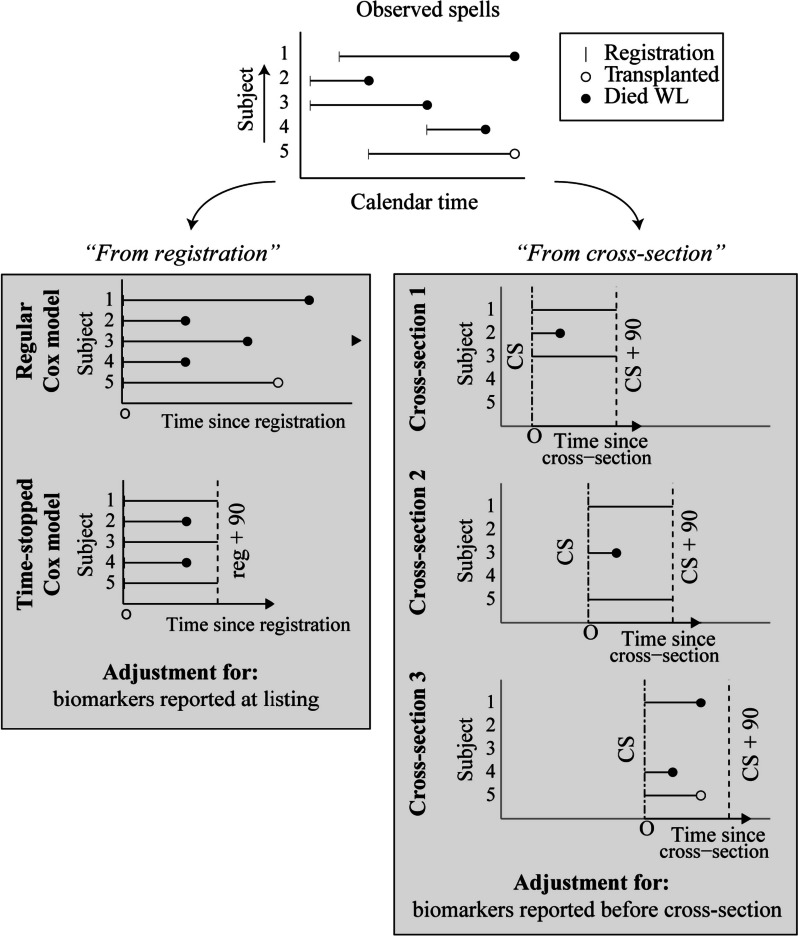
Illustration of the *“from registration”* and *“from cross-section”* approaches to modelling waitlist mortality. For revision of MELD, typically 90-day time-stopped Cox models are used. The *“from registration”* approach (left) uses time since registration as the time scale and adjusts for biomarkers reported at registration. The *“from cross-section”* approach (right*)* models time-until-death from cross-section dates, pre-specified in calendar time. Adjustment is for MELD biomarkers reported before the cross-section date

In revising MELD authors typically re-estimate MELD coefficients *“from registration”*, i.e. using Cox models for 90-day waitlist mortality after registration with adjustment for MELD biomarkers at listing. Coefficients for the MELD scoring system are then commonly obtained by rescaling estimated coefficients $\widehat{\beta }$ to the UNOS-MELD scale by matching quantiles of the linear predictor to quantiles of UNOS-MELD scores (e.g. [6, 9]). This *“from registration”* approach ignores any MELD measurements recorded after registration, as well as patient deaths recorded more than 90 days after registration. We propose to circumvent such waste of statistical information by revising MELD with a *“from cross-section”* approach, and illustrate key differences between the *“from registration”* approach and *“from cross-section”* approach in Fig. 1.

The *“from cross-section”* approach is based on Gong & Schaubel [12], and models the remaining time-until-death from pre-specified calendar-time cross-sections (see right panel, Fig. 1). The Cox model is stratified by cross-section, uses cross-section calendar times as the time origin, and time elapsed since cross-section as the time scale. At each cross-section only patients with an active registration (i.e., without non-transplantable status) are included for analysis, and Cox models adjust only for biomarker information reported *before* the cross-section. We point out that patients waiting at multiple calendar-time cross-sections contribute multiple observations to the Cox model fit (right panel, Fig. 1). Thereby, also waitlist deaths occurring more than 90 days after waitlist registration and biomarker measurements reported while on the waiting list inform revision of MELD “*from cross-section”*.

In this paper, we directly compare revision of MELD *“from registration”* to revision *“from cross-section”*. In revising MELD *“from registration”*, we stratify models by country of listing. For the *“from cross-section”* approach, we use weekly cross-sections from 31–12-2006 to 22–12-2019 and stratify Cox models by country and cross-section. Survival status 90 days after the cross-section date is used as an endpoint, and adjustment at each cross-section is for the last reported MELD biomarker values before the cross-section date.

### Outcome definition

Time-until-waitlist death is modelled with 90-day time-stopped Cox PH models. Delisted patients who die within 90 days of deregistration are treated as if they had died on waitlist exit (as in [9]). Patients who were transplanted/delisted within 90 days are censored at their exit time. Inverse probability censoring weighting (IPCW) is used to correct for selection bias by transplantation/delisting.

### Inverse probability censoring weighting to correct for dependent censoring by transplantation and delisting

Consistent estimation of parameters $\beta$ with a standard Cox PH model requires that the censoring process is independent of survival conditional on adjustment variables. This independent censoring assumption is violated for both the *“from registration”* and *“from cross-section”* approach, as MELD biomarkers reported after listing/cross-section affect patient survival and transplantation/delisting rates while adjustment is exclusively for *historic* values of MELD biomarkers. Gong & Schaubel [12] proposed to correct for dependent censoring by transplantation by weighing patients by the inverse probability of being transplanted between the cross-section date and exit date (IPCW-T weights, T for transplantation). Such probabilities may be estimated with an extended Cox model which uses transplantation status as the outcome (for details, see Supplementary material 1: Appendix B).

We expand in this paper on Gong & Schaubel’s approach by also constructing inverse probability censoring weights for waitlist removal (IPCW delisting (IPCW-D) weights). Under the assumption that delisting and transplantation are conditionally independent, a joint inverse probability censoring weight can then be obtained as the product of IPCW-T and IPCW-D weights (see also [14]). Details on how weights were constructed are included in Supplementary material 1: Appendix B. In this paper, we assess how IPCW affects revised MELD coefficients both *“from registration”* and *“from cross-section”*.

### Adjustment variables, caps, and functional forms

Cox PH models adjusted for variables present in MELD, i.e., the INR, serum creatinine, and serum bilirubin. Spline terms were used to assess whether the relation between log-transformed biomarkers and the mortality rate is approximately linear. Final models adjusted for logarithmic transformations of the biomarkers, with lower and upper limits for biomarkers optimized over regions where violation of log-linearity was visually apparent (as in [6, 9]).

Eurotransplant liver allocation ignores measured creatinine for patients on biweekly dialysis (> 10% of patients) with UNOS-MELD scores calculated as if patients on dialysis had maximum serum creatinine (4.0 mg/dL). We set creatinine to 1.0 mg/dL for patients on biweekly dialysis (leading to $\log(1.0)=0$ MELD points) to also ignore creatinine levels for patients on dialysis in revising MELD. Instead, we adjusted directly for whether the patient receives biweekly dialysis.

### Development and validation cohorts

We planned to assign patients to 70/30% development/validation cohorts based on their listing center, i.e. assign all patients registered within a center to either the development or validation cohort. Such a center-based split allows for structural differences between development and validation cohorts, and thereby enable geographical validation of revised MELD scores.

To enable revision and geographical validation for all ET countries, an approximate 70%/30% center-based split per country was needed. Such a split was feasible for Germany (30.0/70.0%), Belgium (29.9/70.1%), Austria (37.4%/62.6%), and the Netherlands (25.7/74.3%), but not for Hungary (1 center), Slovenia (1 center), and Croatia (1 large center, 2 very small centers). Therefore, Hungarian, Slovenian and Croatian patients, 11% of the total cohort, were split randomly in 70/30% development/validation cohorts.

All models – including models for estimation of inverse probability weights – were fitted on the development cohort only. The validation cohort was used to compare the newly developed score, DynReMELD, to ReMELD and UNOS-MELD.

### Comparison to UNOS-MELD and ReMELD

We revised MELD *“from registration”* and *“from cross-section”* both with and without IPCW. Without IPCW, MELD was also revised with ReMELD’s linear predictor as an offset. This enables assessment of whether revision of MELD on all cirrhotic patients yields a significantly different equation from ReMELD. We define *DynReMELD* as the equation obtained by quantile matching the linear predictor revised *“from cross-section”* with IPCW to quantiles of UNOS-MELD.

We compare discrimination of *DynReMELD* (UNOS-MELD revised with IPCW from cross-section) to UNOS-MELD and ReMELD in the validation cohort with a time-truncated c-index with correction for dependent censoring [15] (see Supplementary material 1: Appendix D for details). This c-index quantifies to what extent patients with a higher score die earlier than patients with a lower score on the ET waiting list. We assess this discrimination using c-indices for two separate prediction tasks, being (i) prediction of time-until-death at listing based on biomarkers reported at listing, and (ii) prediction of time-until-death at calendar-time cross-sections based on the last reported MELD biomarkers. Assessment of calibration for *DynReMELD* is complicated by the fact that models developed with IPCW are counterfactual prediction models, and it is not clear how to assess calibration for such models [16]. We instead chose to report estimates of absolute 90-day survival risks for *DynReMELD* estimated with and without IPCW.

## Results

This study included 13,343 liver waitlist registrations for 13,274 patients^1^ with chronic liver cirrhosis waiting for a first transplant. 107 patients (< 1%) were excluded because they reported impossible MELD biomarker values (e.g., all zeroes). Baseline characteristics for development and validation cohorts are included in Supplementary Table 1.

### Number of MELD scores informing MELD revision

With weekly cross-sections, 8,779 out of 9,288 (95%) patients in the development cohort are active at a cross-section date, thereby inform revision of MELD *“from cross-section”*. The remaining 509 patients (5%) are transplanted/delisted/non-transplantable before a cross-section date is reached (within at most 7 days of listing).

Biomarkers reported after registration are ignored when revising MELD *“from registration”*, but can inform revision of MELD *“from cross-section”*. Table 1 shows that the number of unique MELD scores informing MELD revision increases about sevenfold with a *“from cross-section”* approach, from 9,264 *“from registration”* to 67,433. The number of observed waitlist deaths and event rates also increase substantially with the *“from cross-section”* approach. E.g., *“from cross-section”* the number of included MELD scores between 36 and 40 triples from 456 to 1,248, with 47% of MELD 36–40 patients dying within 90 days *“from cross-section”* compared to only 31% *“from registration”*. The fraction of patients transplanted within 90 days after registration is substantially higher than the fraction of patients transplanted within 90 days after calendar-time cross-sections, both globally and for subgroups of UNOS-MELD scores. This reflects that registration of a patient reflects an intention to transplant by the center of listing.

**Table 1:** Number of UNOS-MELD scores used for the model fit in the “*from registration”* approach, and “*from cross-section*”-approach

Event within 90 days				
	**Dataset**	**# usable MELD scores**	**Death/removed unfit**	**Transplanted**
**Total**				
	From registration	9264	846 (9.1%)	2598 (28.0%)
	From cross-section	67,433	5906 (8.8%)	11,071 (16.4%)
**By UNOS-MELD**				
6–14	From registration	3291	67 (2.0%)	389 (11.8%)
	From cross-section	28,192	454 (1.6%)	1565 (5.6%)
15–24	From registration	4355	382 (8.8%)	1199 (27.5%)
	From cross-section	30,806	2938 (9.5%)	5766 (18.7%)
25–35	From registration	1160	253 (21.8%)	711 (61.3%)
	From cross-section	7187	1922 (26.7%)	3144 (43.7%)
36–40	From registration	458	144 (31.4%)	299 (65.3%)
	From cross-section	1248	592 (47.4%)	596 (47.8%)

### Re-estimated coefficients with Cox models

Leise et al. [6] and Goudsmit et al. [9] derived evidence-based caps for MELD biomarkers by choosing upper and lower biomarker limits such that the log-likelihood of multivariable Cox models is maximal. We followed this procedure and found optimal bounds to be 0.8–2.5 mg/dL for creatinine, 1.0–3.0 for the INR, and 0.6–55 mg/dL for bilirubin (see Supplement material 1: Appendix C for details). Here, we report MELD equations revised *“from registration”* and *“from cross-section”* with these bounds applied. 

#### From registration 

Panel A of Table 2 shows MELD coefficients revised *“from registration”*. The first column shows that parameter estimates are jointly insignificantly different from 0 (${\chi }_{3}^{2} = 4.1, p = 0.25)$ when using ReMELD’s prognostic index as an offset. Insignificance assures us that ReMELD adequately predicts 90-day mortality *“from registration”* for all cirrhotic patients. Coefficients revised without offset are shown without IPCW in column 2, and with weighting in column 3. IPCW changes biomarker coefficients change only slightly (by less than a standard error).

**Table 2:** Comparison of MELD coefficients for different model fits *“from registration*” (panel A) *and “from cross-section”* (panel B), for (1) revision with ReMELD’s prognostic index as an offset, (2) revision without the offset, and (3) revision with IPCW

Panel A—*"From registration"*			
	Time to death from baseline, 90d		
	(1) ReMELD offset	(2) Refitted, no IPCW	(3) Refitted, IPCW
log(creatinine^a^ (mg/dL))	0.10 (0.09)	1.62 (0.01)	1.61 (0.12)
log(bilirubin (mg/dL))	0.03 (0.05)	0.69 (0.05)	0.73 (0.06)
log(INR)	0.12 (0.17)	2.02 (0.17)	2.19 (0.21)
Biweekly dialysis		1.85 (0.12)	1.73 (0.13)
LR Test	4.1 (df = 3)	1482 (df = 4)	1883 (df = 4)
Panel B—*"From cross-section"*			
	Time to waitlist death from cross-section, 90d		
	(1) ReMELD offset	(2) Refitted, no IPCW	(3) Refitted, IPCW
log(creatinine^a^ (mg/dl))	0.40 (0.08)	2.07 (0.08)	2.15 (0.08)
log(bilirubin (mg/dl))	0.22 (0.04)	0.92 (0.04)	0.97 (0.04)
log(INR)	0.04 (0.13)	2.06 (0.13)	2.22 (0.15)
Biweekly dialysis		1.87 (0.12)	1.86 (0.12)
LR Test	801 (df = 3)	22 197 (df = 4)	26 933 (df = 4)

^a^creatinine set to 1.0 if on dialysis

#### From cross-section

Panel B of Table 2 shows MELD coefficients revised *“from cross-section”*. The first column shows that coefficients are jointly significantly different from 0 with ReMELD offset ($${\chi }_{3}^{2}=801,p<0.001$$). Hence, ReMELD does not adequately predict 90-day mortality from cross-section. Estimated coefficients suggest ReMELD underestimates coefficients for creatinine ($$z=5.2, p <0.001)$$ and bilirubin ($$z=6.1,p<0.001$$), but not the INR ($$z=0.3,p=0.76$$). IPCW again appears to increase MELD biomarker coefficients slightly (less than a standard deviation, see column 2 and 3).

Supplementary Table 2 shows relative weights put on MELD components by the equation revised from cross-section with IPCW, UNOS-MELD and ReMELD. The weights, defined by Sharma et al. [5], quantify the increase in MELD score due to a one-standard deviation increase in the biomarker relative to a one-standard deviation increase in all biomarkers. These weights confirm that the refitted equation puts more weight on bilirubin (41%) than ReMELD (37%) or UNOS-MELD (36%), and puts less weight on the INR (28% vs. 32% for UNOS-MELD and 34% for ReMELD).

### Definition of the DynReMELD score

Quantile matching of UNOS-MELD to the linear predictor revised *“from cross-section”* with IPCW (Table 2, panel B) yielded the following equation for *DynReMELD:*

$$9.12\times {\text{log}}\left(\mathrm{creatinine }\left({\text{mg}}/{\text{dl}}\right)\right)+4.14\times {\text{log}}\left(\mathrm{bilirubin }\left({\text{mg}}/{\text{dl}}\right)\right)+9.42\times {\text{log}}\left({\text{INR}}\right)+8.50,$$

with creatinine bounded to 0.8–2.5 mg/dL, bilirubin to 0.6–55 mg/dL, and the INR to 1.0–3.0. In line with existing clinical implementations of MELD scores, we calculate DynReMELD by setting creatinine to the upper cap (2.5 mg/dL) for patients on dialysis. This is relatively harmless despite the fact that risk equations were estimated with a separate parameter for biweekly dialysis, as the creatinine level required to attain equal priority as biweekly dialysis is ${\text{exp}}\left(1.86/2.15\right) \approx 2.4$ mg/dL (Table 2, Panel B, third column).

### Predictive performance

Table 3 shows estimated time-truncated c-indices for UNOS-MELD, ReMELD and DynReMELD, for (a) predicting 90-day waitlist survival at listing based on biomarkers reported at listing, and (b) predicting 90-day waitlist survival at calendar-time cross-sections, based on last reported biomarkers (see Table 3). These c-indices quantify the fraction of comparable pairs of patients where the patient with highest predicted risk had shorter survival (perfect prediction yielding a c-index of 1).

**Table 3:** c-indices at 90 days after listing with bootstrapped standard errors in brackets

	Development	Validation
**Predicting time-until-death at listing, based on biomarkers at listing**		
UNOS-MELD	0.8494 (0.008)	0.8637 (0.011)
ReMELD	0.8503 (0.008)	0.8623 (0.011)
DynReMELD	0.8523^†^ (0.008)	0.8641 (0.011)
**Predicting time-until-death at calendar-time cross-sections, based on last reported biomarkers**		
UNOS-MELD	0.8099 (0.002)	0.7855 (0.004)
ReMELD	0.8203^***^ (0.002)	0.7879 (0.004)
DynReMELD	0.8217^***†††^ (0.002)	0.7895^***††^ (0.004)

***p < 0.001, compared to UNOS-MELD

^†^*p* < 0.05;††*p* < 0.01;†††*p* < 0.001, compared to ReMELD

The first panel shows c-indices evaluated for predicting 90-day waitlist survival at listing based on biomarkers reported at listing for UNOS-MELD, ReMELD and DynReMELD. Point estimates appear to slightly favor DynReMELD, but bootstrapped pairwise differences are not statistically significant. The second panel shows that DynReMELD outperforms ReMELD and UNOS-MELD when predicting 90-day waitlist survival based on patient’s last reported biomarkers, with DynReMELD attaining higher c-indices ($p<0.001$) in both development and validation cohorts. In the validation cohort the c-index of DynReMELD (0.7895) is approximately 0.0040 higher than UNOS-MELD (0.7855), and approximately 0.0015 higher than ReMELD (0.7879).

### Estimated absolute survival risks per score

This section reports absolute 90-day mortality risks for UNOS-MELD and DynReMELD estimated *“from cross-section”*. Estimation of mortality risks *“from cross-section”* is complicated by the fact that most individuals contribute multiple, correlated observations to the Cox model. In principle, dependence can be broken by reporting cross-section specific estimates of 90-day waitlist survival, but such estimates are imprecise. To partially break the dependence, we chose to estimate 90-day survival on a data set which included for each reported set of MELD biomarkers only the first cross-section at which the corresponding patient had an active waitlist registration. Table 4 shows 90-day mortality risks estimated in this way.

**Table 4:** Eurotransplant mortality equivalents per score, and estimates of 90-day mortality risks per score. 90-day mortality risks were estimated with Cox models fitted *‘from cross-section’*, adjusting for the point score

Estimated 90-day mortality risks				
**Score**	**UNOS-MELD** **(no IPCW)**	**UNOS-MELD** **(with IPCW)**	**DynReMELD** **(no IPCW)**	**DynReMELD** **(with IPCW)**
20	0.103 [0.098–0.108]	0.122 [0.117–0.127]	0.097 [0.092–0.101]	0.113 [0.108–0.118]
22	0.149 [0.142–0.155]	0.179 [0.171–0.186]	0.145 [0.139–0.151]	0.173 [0.166–0.180]
24	0.212 [0.202–0.221]	0.258 [0.247–0.270]	0.214 [0.205–0.224]	0.260 [0.249–0.271]
25	0.251 [0.239–0.263]	0.308 [0.294–0.322]	0.259 [0.247–0.271]	0.315 [0.301–0.329]
26	0.297 [0.282–0.311]	0.365 [0.348–0.382]	0.310 [0.295–0.325]	0.379 [0.361–0.396]
28	0.407 [0.385–0.428]	0.498 [0.473–0.522]	0.435 [0.413–0.457]	0.529 [0.503–0.553]
29	0.470 [0.444–0.495]	0.572 [0.544–0.599]	0.508 [0.482–0.533]	0.612 [0.583–0.639]
30	0.538 [0.508–0.566]	0.649 [0.617–0.678]	0.585 [0.555–0.613]	0.696 [0.664–0.725]
31	0.609 [0.576–0.640]	0.725 [0.691–0.755]	0.664 [0.632–0.694]	0.777 [0.744–0.805]
32	0.681 [0.645–0.714]	0.796 [0.762–0.825]	0.742 [0.708–0.772]	0.848 [0.817–0.874]
33	0.751 [0.714–0.784]	0.859 [0.827–0.885]	0.814 [0.780–0.842]	0.907 [0.880–0.927]
34	0.816 [0.780–0.846]	0.910 [0.883–0.931]	0.875 [0.845–0.900]	0.949 [0.929–0.964]
35	0.872 [0.839–0.899]	0.949 [0.927–0.964]	0.925 [0.899–0.943]	0.977 [0.963–0.985]
36	0.918 [0.890–0.940]	0.974 [0.960–0.984]	0.959 [0.941–0.972]	0.991 [0.984–0.995]
37	0.953 [0.930–0.968]	0.989 [0.980–0.994]	0.981 [0.969–0.989]	0.997 [0.994–0.999]
39	0.989 [0.979–0.994]	0.999 [0.997–1.000]	0.998 [0.995–0.999]	1.000 [1.000–1.000]
40	0.996 [0.991–0.998]	1.000 [0.999–1.000]	0.999 [0.998–1.000]	1.000 [1.000–1.000]

Table 4 shows that inverse probability censoring weighting increases estimates of absolute 90-day mortality risks by almost 10 percentage points. Failing to correct for informative censoring thus results in mortality equivalents which understate the counterfactual mortality risk. This is of interest to Eurotransplant, as mortality equivalents are used by Eurotransplant in liver allocation for assigning exception points to non-cirrhotic patients.

In estimating 90-day mortality risks *“from cross-section”* we allowed patients with multiple reported MELD scores to contribute multiple observations. Dependence between such observations can bias estimated 90-day mortality risks. Reassuring is that point estimates of 90-day mortality risks *“from registration”* (Supplementary Table 3) generally differ by less than 5 percentage points to estimates “*from cross-section”*.

Another potential issue is that estimated 90-day mortality risks may be biased in case the proportional hazards assumption is violated for MELD [17] proposed to avoid the proportional hazards assumption by estimating 90-day mortality risks with the Kaplan–Meier estimator with stratification on the MELD score, with removal of the dependence between repeated observations on the same individual by including for analysis only the first time a candidate reaches a particular MELD score. Supplementary Fig. 1 shows that 90-day mortality risks estimated with this alternative approach are similar to estimates based on the Cox model, suggesting that the proportional hazards assumption induces minimal bias.

## Discussion

Prior literature revised MELD with liver waitlist candidate data *“from registration”* (e.g., [7–9]), ignoring biomarker measurements after registration and waitlist deaths occurring more than 90 days after registration. We modelled waitlist mortality from calendar-time cross-sections, based on Gong & Schaubel [12], to avoid such waste of statistical information in revising MELD. Moreover, we assessed how correction for selection bias by transplantation/delisting with inverse probability censoring weighting affected revision of MELD.

We showed that the *“from cross-section”* approach uses waitlist registry data substantially more efficiently, with the number of waitlist deaths and MELD scores informing revision of MELD increasing sevenfold compared to revision *“from registration”*. DynReMELD, the score obtained by quantile matching UNOS-MELD to the risk equation developed *“from cross-section”* with IPCW, attains significantly higher c-indices than ReMELD and UNOS-MELD in a geographical validation cohort for predicting remaining time-until-death based on last reported MELD biomarkers ($p<0.001$). This is important for Eurotransplant, since Eurotransplant liver allocation prioritizes candidates based on their last reported MELD scores (and not MELD at listing). In magnitude improvements in c-indices (0.0015 compared to ReMELD, and 0.0040 compared to UNOS-MELD) are comparable to the addition of serum sodium to ReMELD (approx. delta c-index of 0.0030) [9] and serum albumin to MELD 3.0 (delta c-index of 0.0028) [8]. MELD revision from cross-section with IPCW can thus improve urgency-based risk stratification. Our results suggest that the improvement is due to modelling time-remaining-until-death from cross-sections and not IPCW, as IPCW changed estimated coefficients only slightly.

We believe the main reason why DynReMELD outperforms ReMELD in validation is that revision *‘from cross-section’* uses ET registry data substantially more efficiently than revision *‘from registration’*, as the latter method only uses MELD biomarkers reported at listing and the first 90-days of waitlist survival. This raises the question whether revision *‘from registration’* cannot also be improved upon by using available registry data more efficiently. In principle, MELD biomarkers could be used more efficiently by adjusting for MELD biomarkers as time-varying covariates in the extended Cox model. However, problems would arise when using such models for prediction; this would require knowledge on the complete future trajectories of MELD biomarkers over time at the moment of prediction [18]. Follow-up data could be used more efficiently by not restricting revision *‘from registration’* to the first 90-days after listing. However, we found that this leads to issues with the proportional hazards assumption for MELD biomarkers.

We also assessed how revision of MELD *“from cross-section”* and IPCW affected estimates of absolute 90-day waitlist mortality risks for UNOS-MELD and DynReMELD. Revision *“from cross-section”* does not meaningfully change estimated 90-day mortality risks, with risks estimated *“from cross-section”* differing by less than 5 percent points from risks estimated *“from registration”*. Mitigation of selection bias with IPCW did increase estimated 90-day waitlist mortality risks for both UNOS-MELD and DynReMELD by 10 percentage points. Ignoring that censoring by transplantation/delisting is informative may thus underestimate 90-day mortality equivalents, which is potentially problematic as Eurotransplant uses such estimates to assign priority points for non-cirrhotic patients.

Within the Eurotransplant member countries there are currently 39 active liver transplantation centres. These centres differ structurally in terms of patient populations due to differing national guidelines on waitlist eligibility, differ in liver transplantation volumes, and have different graft offer acceptance criteria for example for acceptance of donors of marginal quality. A strength of our study is that we assigned candidates to either the development or validation cohort based on their center of listing, which means that the predictive performance of DynReMELD was evaluated in a cohort independent from the centres on which DynReMELD was developed.

A limitation of our work is that adjustment in revision of MELD *“from cross-section”* was for last reported MELD biomarkers before the cross-section date. Eurotransplant uses these same measurements for allocation, but they may be dated representations of a patient’s health status. Alternatively, one could model the evolution of MELD biomarkers over time with linear mixed models, and adjust at each cross-section for best linear unbiased predictions (BLUP) of biomarkers at the cross-section time. This BLUP approach was first proposed by Maziarz et al. [19] for landmarking, a statistical technique which bears similarities to Gong & Schaubel’s approach. We did not use a BLUP approach for this paper, since irregular spacing of MELD measurements complicates modelling the biomarker process and deployment of BLUP models would be practically challenging for Eurotransplant. Moreover, MELD scores for patients with significant 90-day mortality risks are rarely dated as Eurotransplant requires frequent recertification for sicker patients. E.g., the average age of MELD scores at cross-section is 12 days for patients with MELD 20–25 (corresponding to an approximate 10% 90-day mortality risk), and 3 days old for MELD > 25 (corresponding to a > 25% mortality risk).

Another limitation of our work is that DynReMELD was based only on bilirubin, creatinine and the INR, whereas other allocation scores exist which additionally include serum sodium (MELD-Na) and serum albumin (MELD 3.0). Future work could focus on revising these UNOS-MELD alternatives *‘from cross-section’.* This was not pursued in this paper, as serum sodium and albumin are not routinely reported for most Eurotransplant patients.

### Supplementary Information

**Supplementary material 1.**

## Data Availability

Data used for this article are not publicly accessible. Patients have given consent that Eurotransplant can use their personal health data for allocation development, but not permission to make the data broadly accessible. Parties interested in the data may send a study request to the Eurotransplant Liver and Intestine Advisory Committee to obtain anonymized versions of the dataset used for the article. Code is available upon request from the corresponding author.

## Abbreviations

ET: Eurotransplant
INR: International Normalized Ratio
IPCW: Inverse probability censoring weighting
NT: Non-transplantable: status used in Eurotransplant to indicate a patient is (temporarily) unavailable for transplantation
MELD: Model for End-stage Liver Disease
MELD-Na: Model for End-stage Liver Disease sodium
Cox PH: Cox proportional hazards
ReMELD: Refitted MELD for Eurotransplant
TRIPOD: Transparent Reporting of a multivariable prediction model for Individual Prognosis Or Diagnosis
UNOS: United Network for Organ Sharing

## Footnotes

1 A small group of patients is removed from the waiting list without transplant, but later re-registered.
