# Supplementary material for: Revising model for end-stage liver disease from calendar-time cross-sections with correction for selection bias
Source: BMC Med Res Methodol. 2024 Feb 28;24:51. doi: 10.1186/s12874-024-02176-8 (PMC10900649; doi:10.1186/s12874-024-02176-8)
Supplement: Supplementary file 1 — Supplementary material 1. [file 12874_2024_2176_MOESM1_ESM.docx]

Supplementary materials

1. – Supplementary materials

Supplementary Table 1 - Baseline characteristics and observed waitlist outcomes for patients included in this study

|  | **Development (N=9,288)** | **Validation (N=4,055)** | **P-value** |
| --- | --- | --- | --- |
| **UNOS-MELD at listing** |  |  |  |
| Mean (Q1-Q3) | 21.0 (13.0-28.0) | 20.3 (12.0-28.0) | <0.001 |
| **Creatinine (mg/dl) at listing** |  |  |  |
| Mean (Q1-Q3) | 1.54 (0.810-1.75) | 1.46 (0.803-1.60) | 0.001 |
| **Bilirubin (mg/dl) at listing** |  |  |  |
| Mean (Q1-Q3) | 7.53 (1.63-8.54) | 7.06 (1.43-8.00) | 0.009 |
| **INR** |  |  |  |
| Mean (Q1-Q3) | 1.79 (1.29-2.00) | 1.75 (1.21-1.91) | 0.024 |
| **Biweekly dialysis** |  |  |  |
| Yes | 1096 (11.8%) | 504 (12.4%) | 0.318 |
| No | 8192 (88.2%) | 3551 (87.6%) |  |
| **Patient sex** |  |  |  |
| Male | 6226 (67.0%) | 2761 (68.1%) | 0.239 |
| Female | 3062 (33.0%) | 1294 (31.9%) |  |
| **Age at listing** |  |  |  |
| Mean (Q1-Q3) | 53.8 (49.0-61.0) | 54.4 (49.0-61.0) | <0.001 |
| **Cirrhosis aetiology** |  |  |  |
| Alcoholic | 4949 (53.3%) | 2292 (56.5%) | <0.001 |
| Autoimmune/cryptogenic | 1596 (17.2%) | 619 (15.3%) |  |
| Hepatitic | 1712 (18.4%) | 528 (13.0%) |  |
| Metabolic/other/unknown | 669 (7.2%) | 484 (11.9%) |  |
| NAFLD | 362 (3.9%) | 132 (3.3%) |  |
| **Event** |  |  |  |
| Died WL (or within 90 days  of removal) | 2265 (24.4%) | 980 (24.2%) | <0.001 |
| Transplanted | 5068 (54.6%) | 2105 (51.9%) |  |
| Removed (other) | 700 (7.5%) | 278 (6.9%) |  |
| Recovered | 610 (6.6%) | 327 (8.1%) |  |
| Censored (waiting by 31-12-2019) | 645 (6.9%) | 365 (9.0%) |  |
|  |  |  |  |

Supplementary Table 2– Relative weights put on bilirubin, creatinine and INR by UNOS-MELD, ReMELD and DynReMELD. Relative weights were calculated $w_{i}=\frac{\beta_{i}SD_{i}}{\sum_{j} \beta_{j}SD_{j}}$, where $\beta_{i}$ is the coefficient on biomarker $i$ and $SD_{i}$ its standard deviation in the development cohort.

| Biomarker | **s.d.** | **UNOS-MELD** | **ReMELD** | **DynReMELD** |
| --- | --- | --- | --- | --- |
| Bilirubin (mg/dl) | 0.85 | 36% | 37% | 41% |
| Creatinine (mg/dl) | 0.29 | 32% | 29% | 31% |
| INR | 0.26 | 32% | 34% | 28% |

Supplementary Table 3 – Estimates of absolute 90-day mortality risks *“from registration”* and *“from cross-section”*. Reported risks were estimated without IPCW.

|  |  | Estimated 90-day mortality risks | | | |
| --- | --- | --- | --- | --- | --- |
| **Score** | **Mrt. Eq.** | **DynReMELD from registration** | **DynReMELD from cross-section** | **UNOS-MELD from registration** | **UNOS-MELD from cross-section** |
| 20 | 10% | 0.125 [0.116-0.132] | 0.097 [0.092-0.101] | 0.136 [0.127-0.144] | 0.103 [0.098-0.108] |
| 22 | 15% | 0.176 [0.166-0.186] | 0.145 [0.139-0.151] | 0.187 [0.176-0.197] | 0.149 [0.142-0.155] |
| 24 | 20% | 0.247 [0.233-0.260] | 0.214 [0.205-0.224] | 0.254 [0.240-0.267] | 0.212 [0.202-0.221] |
| 25 | 25% | 0.290 [0.274-0.305] | 0.259 [0.247-0.271] | 0.294 [0.278-0.309] | 0.251 [0.239-0.263] |
| 26 | 30% | 0.339 [0.321-0.356] | 0.310 [0.295-0.325] | 0.339 [0.321-0.356] | 0.297 [0.282-0.311] |
| 28 | 35% | 0.453 [0.429-0.476] | 0.435 [0.413-0.457] | 0.443 [0.420-0.466] | 0.407 [0.385-0.428] |
| 29 | 45% | 0.518 [0.491-0.543] | 0.508 [0.482-0.533] | 0.502 [0.476-0.527] | 0.470 [0.444-0.495] |
| 30 | 50% | 0.586 [0.556-0.613] | 0.585 [0.555-0.613] | 0.564 [0.535-0.591] | 0.538 [0.508-0.566] |
| 31 | 55% | 0.655 [0.623-0.684] | 0.664 [0.632-0.694] | 0.627 [0.596-0.657] | 0.609 [0.576-0.640] |
| 32 | 60% | 0.724 [0.690-0.753] | 0.742 [0.708-0.772] | 0.691 [0.657-0.721] | 0.681 [0.645-0.714] |
| 33 | 70% | 0.788 [0.755-0.817] | 0.814 [0.780-0.842] | 0.753 [0.718-0.783] | 0.751 [0.714-0.784] |
| 34 | 75% | 0.847 [0.815-0.873] | 0.875 [0.845-0.900] | 0.810 [0.776-0.839] | 0.816 [0.780-0.846] |
| 35 | 80% | 0.896 [0.868-0.919] | 0.925 [0.899-0.943] | 0.862 [0.830-0.888] | 0.872 [0.839-0.899] |
| 36 | 85% | 0.935 [0.912-0.953] | 0.959 [0.941-0.972] | 0.905 [0.877-0.927] | 0.918 [0.890-0.940] |
| 37 | 90% | 0.963 [0.946-0.975] | 0.981 [0.969-0.989] | 0.939 [0.916-0.956] | 0.953 [0.930-0.968] |
| 39 | 95% | 0.992 [0.985-0.996] | 0.998 [0.995-0.999] | 0.981 [0.968-0.989] | 0.989 [0.979-0.994] |
| 40 | 100% | 0.997 [0.993-0.999] | 0.999 [0.998-1.000] | 0.991 [0.983-0.995] | 0.996 [0.991-0.998] |

Supplementary Figure 1 – Comparisons of estimated relation between MELD scores and the absolute 90-day mortality risk. Curved lines show a smoothed relation based on proportional hazards model, estimated from cross-sections with IPCW. Dots present estimates of the absolute 90-day mortality risks by Kaplan-Meier, stratified by the score. Standard errors were estimated as in Ruth et al. (with the method of Xie and Liu).


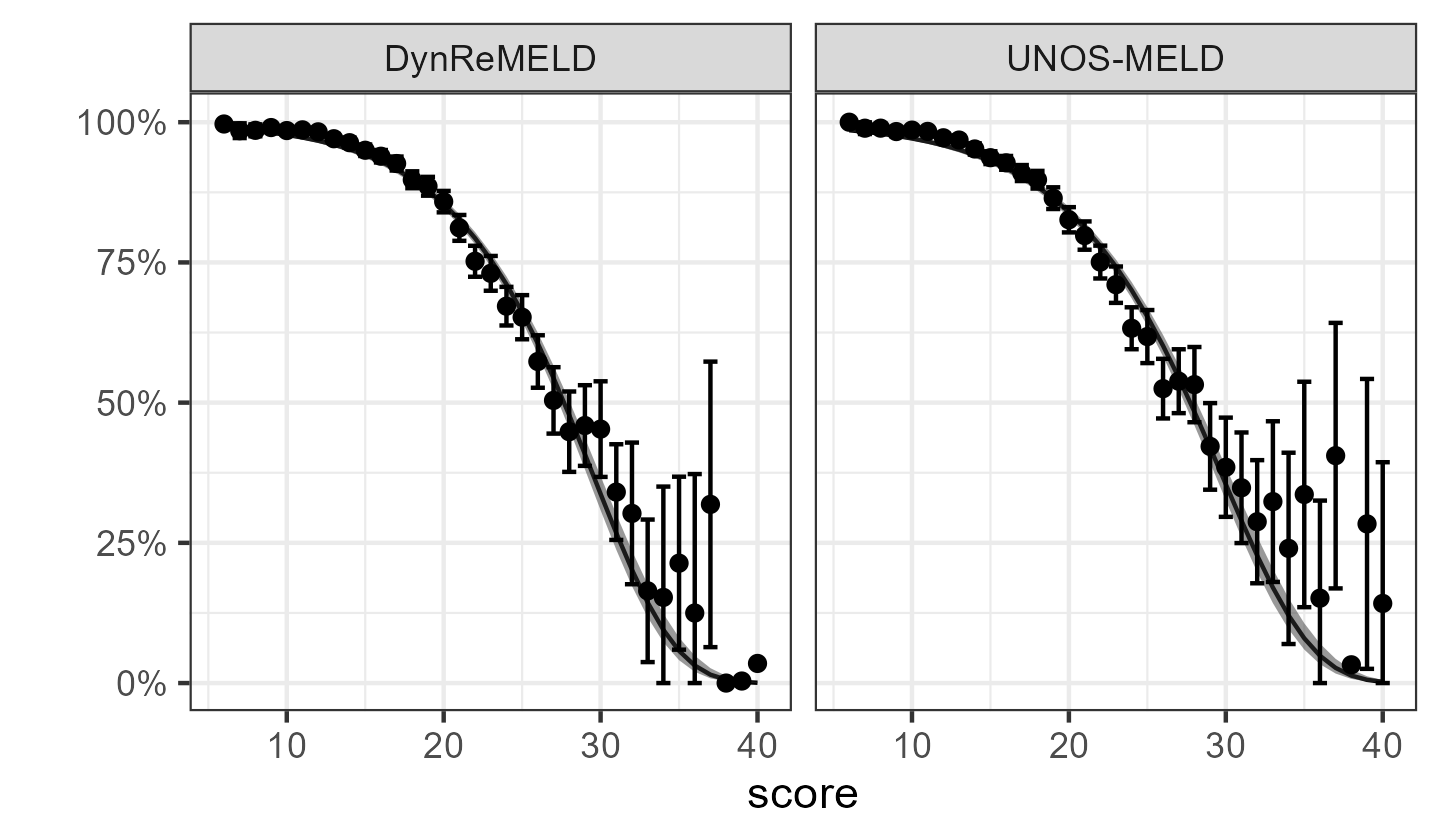


1. – Procedure to obtain evidence-based caps

Figure B1 shows relations between the relative mortality rate and bilirubin, creatinine (without dialysis), and the INR, contrasting the “*from-registration”* with the *“from cross-section”* approach. The plots confirm that the relation between MELD biomarkers and the relative mortality rate is approximately log-linear for most of the domain (i.e. a straight line).

For bilirubin, log-linearity appears reasonable for most of the domain except values lower than 1.0 mg/dL. The effect of bilirubin plateaus off at 20 mg/dL with a *“from registration”* approach, but does not plateau with the *“from cross-section”* approach. For creatinine, log-linearity plateaus off for values higher than $\pm$3.0 mg/dL with both approaches. For INR, a kink appears over 3.0 mg/dL, after which confidence bands are wide. Confidence bands are also wide for INRs below 1.0.

Figure B1 - Relations between the relative mortality rate and MELD biomarkers, separately for the “from registration” and “from cross-section” approaches. Relations were estimated with multivariable Cox PH models in the development cohort, with penalized spline terms for MELD biomarkers with 4 degrees of freedom, with boundary knots placed at the 2.5% and 97.5% percentiles.


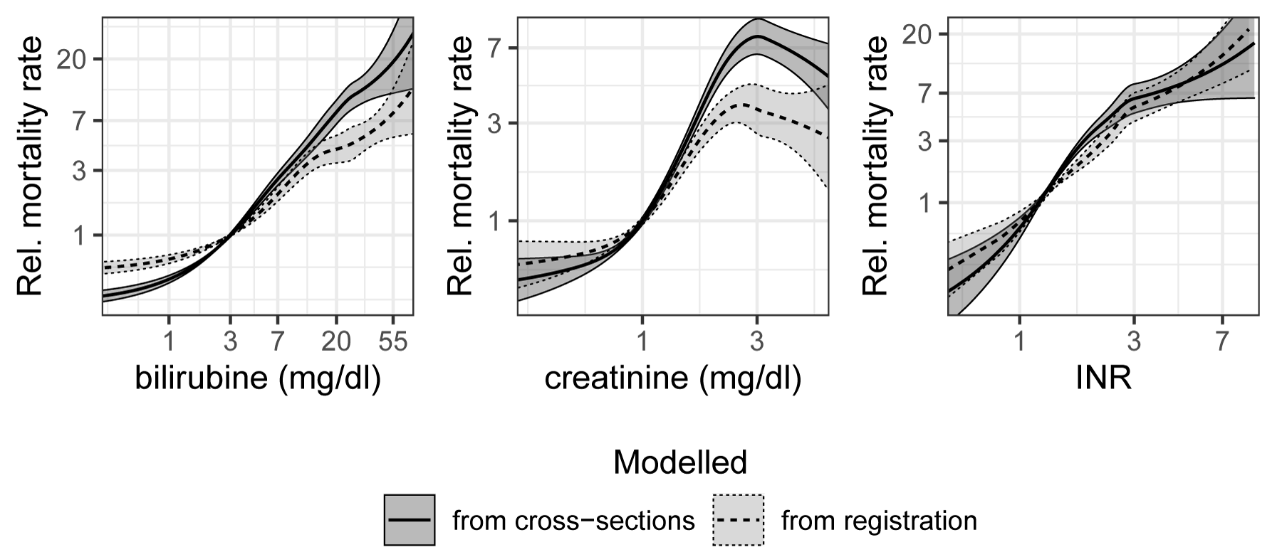


Caps for MELD biomarkers were optimized by choosing upper limits and lower limits which maximized the log-likelihood of multivariable Cox models (as in (6,9)). Figure B2 summarizes the results of this procedure. For creatinine, optimal bounds were found to be 0.8 and 2.5 mg/dL. For INR, a lower and upper bound of 1.0 and 3.0 were optimal. For bilirubin, 0.6-55 mg/dL was optimal.

Figure B2 – Heatmaps for the log-likelihood for combinations of lower and upper bounds for creatinine (mg/dL), INR, and bilirubin (mg/dL). Stars represent the optimal caps in development data, dots represent the caps of Goudsmit et al. (2020).


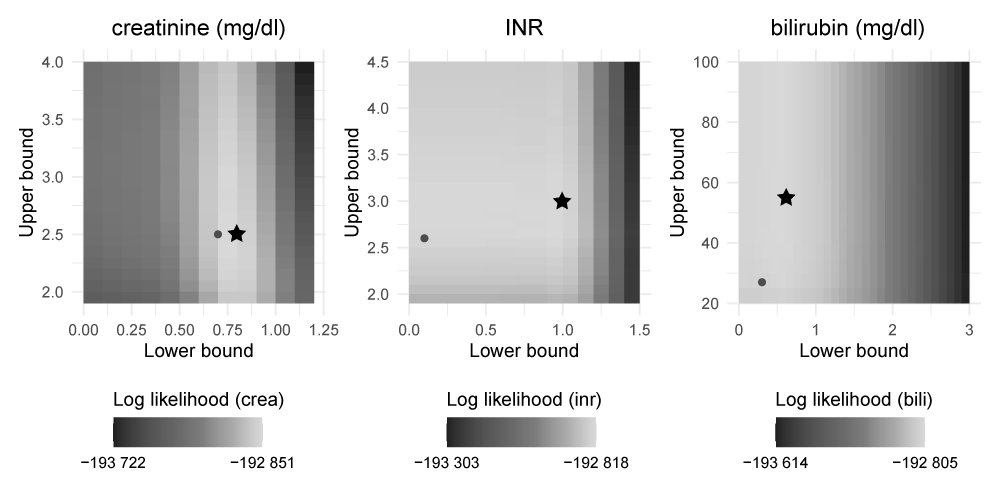


Optimal bounds for creatinine (0.8-2.5 mg/dL) are similar to ReMELD’s (0.7-2.5 mg/dL), with the upper bound slightly lower than the upper bound proposed for UNOS (3.0 mg/dL) [8], [12]. For INR, optimal bounds (1.0–3.0) are higher than proposed for ReMELD (0.1-2.6), but equal to those proposed for UNOS (1.0-3.0). Optimal bounds for bilirubin (0.6-55 mg/dL) are slightly higher than ReMELD (0.3-27 mg/dL). The found bilirubin bounds are better aligned with those proposed for UNOS (lower bound of 1.0 only).

1. – Definition of inverse probability weights

## Inverse probability censoring weighting for transplantation

Here, we introduce notation to explain how Gong & Schaubel derive weights to adjust for dependent censoring. We apply the same procedure to derive weights for waitlist removal. Let $R_{i}$ denote the registration date in calendar time for patient $i$, and $r$ denote the time elapsed since patient $i^{'}s$ registration time $R_{i}$. Each patient has a waitlist death time ($D_{i}$), removal/censoring time $\left( C_{i} \right)$, and transplantation time $T_{i},$ all defined with $R_{i}$ as the time origin. In practice, we only observe the minimum $X_{i}=\min\left( D_{i},C_{i},T_{i} \right)$.

After registration, the patient may become (temporarily) non-transplantable. To account for this, let $A_{i}\left( r \right)$ denote whether patient $i$ has an active registration $r$ time units after registration, i.e. $A_{i}\left( r \right)$ = 1 only if patient $i$is transplantable at time $R_{i}+r.$ In addition, updated covariate information (e.g. MELD scores) may be reported for patient $i$. Denote with $Z_{i}\left( r \right)$ all covariate history reported up to $r$ time units after registration for patient $i.$ Note that this covariate history can consist of observed covariates and other summaries of treatment eligibility history ($A_{i}\left( r \right)$).

The key idea behind Gong & Schaubel’s framework is to introduce a series of cross-section dates ($CS_{1},\ldots CS_{K})$, and model the mortality hazard from each cross-section onwards for patients with an active registration at cross-section date $CS_{k}$. Models are partly conditional, i.e. they adjust for covariate history observed prior to $CS_{k}$. The time scale used is thus the time elapsed since cross-section date $CS_{k}$, denoted by $s.$ For notational convenience, it is helpful to define the time registered for patient $i$ until cross-section $k$ by $S_{ik}$, i.e. $S_{ik}:=CS_{k}-R_{i}.$ Gong & Schaubel’s approach can then be represented with the following hazard model

$\lambda_{ik}^{D}\left( s \right)=A_{i}\left( S_{ik} \right)\lambda_{0k}^{D}\left( s \right)\exp\left\{ \boldsymbol{\beta}_{\boldsymbol{0}}^{'}\boldsymbol{Z}_{\boldsymbol{i}}\left( S_{ik} \right) \right\},\quad s>0 (2)$,

where $A_{i}\left( S_{ik} \right)$ indicates patients are active at the cross-section, $\lambda_{0k}^{D}\left( s \right)$ is a baseline hazard stratified by cross-section, and $Z_{i}\left( S_{ik} \right)$ is patient $i$’s covariate history observed before cross-section date $CS_{k}$.

Direct estimation of (2) through Cox regression results in biased $\hat{\beta_{0}}$, since covariate information (e.g., MELD) reported after cross-section date $CS_{K}$ may still affect the probability of transplantation and waitlist mortality after $CS_{k}$. To correct for this, Gong & Schaubel propose to weigh spells observed from cross section $CS_{k}$ to time $r$ by the inverse conditional probability of remaining on the waiting list up to time $r$, i.e.

$$W_{ik}\left( r \right)=\left[ P\left( T_{i}>r | T_{i}>S_{ik},Z_{i}\left( t \right),t\leq r \right) \right]^{-1}=\left[ \frac{P\left( T_{i}>r | Z_{i}\left( t \right),t\leq r \right)}{P\left( T_{i}>S_{ik} | Z_{i}\left( t \right),t\leq S_{ik} \right)} \right]^{-1}.$$

Gong & Schaubel refer to this weight as the “type A” weight. Note that this weight is only defined as if the conditional probability of being transplanted between the cross-section and $r$ is strictly larger than 0, an assumption known as positivity.

Under the assumption that we can adjust for all variables affecting both transplantation and survival, i.e. no unmeasured confounding, IPCW can be interpreted to construct a “pseudo-population” which would have been observed if transplantation did not exist. Estimation of $\beta_{0}$ through Cox regression on the weighted population therefore recovers counterfactually interpretable parameters. Construction of this pseudo-population requires weights to be estimated. To this end, Gong & Schaubel propose the following treatment hazard model:

$$\lambda_{i}^{T}\left( r | Z_{i}\left( r \right) \right)=A_{i}\left( r \right)\lambda_{0}^{T}\left( r \right)\exp\left\{ \boldsymbol{\theta}_{\boldsymbol{0}}^{'}\boldsymbol{Z}_{\boldsymbol{i}}\left( r \right) \right\} (2).$$

This treatment hazard model use time since registration ($r)$as the time scale, and adjusts for time-varying covariate information ($Z_{i}\left( r \right)$). Using the definition of the hazard rate, one can show that the type A weight reduces to

$$\begin{aligned} W_{ik}(r)&=\left[ \frac{P\left( T_{i}>r | Z_{i}\left( t \right),t\leq r \right)}{P\left( T_{i}>S_{ik} | Z_{i}\left( t \right),t\leq S_{ik} \right)} \right]^{-1}=\exp\left[ \int_{S_{ik}}^{r} A_{i}(u)\lambda_{0}^{T}(u)\exp\left\{ \theta_{0}^{'}Z_{i}(u) \right\} \right] \\ &=\exp\left[ \Lambda_{i}^{T}(r)-\Lambda_{i}^{T}\left( S_{ik} \right) \right], \end{aligned}$$

where $\Lambda_{i}^{T}\left( r \right)=\int_{0}^{r} \lambda_{i}^{T}\left( u | Z\left( u \right) \right)du$ is the cumulative hazard of transplantation.

The type A weight allows for unbiased estimation of $\beta_{0}$ under no unmeasured confounding and positivity. However, since $W_{ik}\left( r \right)$ is an inverse probability weight, it is greater than or equal to 1 for all individuals and cross-sections. This can result in instabilities when conditional probabilities become small. To avoid this, Gong & Schaubel also propose to stabilize the type A weight by a partial conditional estimate of the conditional probability of being transplanted, i.e. stabilize $W_{ik}\left( r \right)$ with

$$P\left( T_{i}>r | Z_{i}\left( S_{ik} \right),t\leq r \right).$$

Gong & Schaubel attain an estimate of this probability using the following partly conditional treatment hazard model,

$$\lambda_{ik}^{T}\left( s \right)=A_{ik}\left( s \right)\lambda_{0k}^{T}\left( s \right)\exp\left\{ \theta_{0}^{'}Z_{i}\left( S_{ik} \right) \right\} (3)$$

Note that this model is partly conditional and uses time since cross-section ($s)$ as the time-scale. Gong & Schaubel confirm with simulations that empirically the type B weight results in smaller standard errors than the type A weight. In this paper, thus type B weights are used.

A graphical summary of how the type A and type B weights are calculated is shown in Figure C1 below.


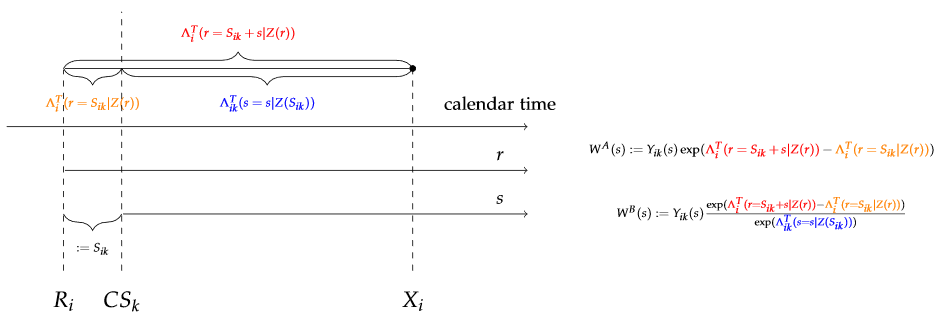


Figure C1 – Figure demonstrating how inverse probability censoring weights (IPCW) are calculated for a subject $i$. Subject $i$ is registered at $R_{i}$, is active at cross-section date $CS_{k}$, and experiences an event at time $X_{i}$. Since subject $i$ has an active registration at $CS_{k}$, this subject contributes an observation to the data set. To correct for dependent censoring, the spell is weighted by the inverse probability that patient $i$ is transplanted between $CS_{k}$ and $X_{i}$, controlling for time-varying $Z\left( r \right)$ (type A weight). To this end, cumulative hazards treatment hazards are estimated from registration $R_{i}$ to $CS_{k}$, and $R_{i}$ to $X_{i}$. The type A weight is the inverse probability of being transplanted before $X_{i}$, conditional on not being transplanted up to $CS_{k}$. It is thus strictly greater than 1, thereby unstabilized. Gong & Schaubel propose to normalize the type A weight by the conditional probability of comparable subjects in cross-section $k$ experiencing an event between $CS_{k}$ and $X_{i}$, conditional on the time-frozen covariate information $Z\left( S_{ik} \right).$

For the treatment hazard model, we adjust for a broad set of confounders since IPCW relies on a no-unmeasured confounding assumption. Patient factors adjusted for are sex, blood group, weight, listing country, and age at listing. Clinical variables adjusted for are whether the patient has a downgraded MELD, is simultaneously listed for a kidney, and the percentage of time a patient has been non-transplantable (too good/too bad/other). We directly adjust for MELD rather than MELD components, since Eurotransplant allocates based on MELD. Since allocation is a national affair, we also interact MELD with the patient country.

## IPCW for transplantation and waitlist delisting

The same approach can be applied to derive type B-weights for waitlist delisting. Under the assumption that waitlist delisting and transplantation are conditionally independent, an “overall” censoring weight can be derived as the product between “type B” weights for censoring and transplantation. We use this joint weight to correct for dependent censoring by transplantation and waitlist removal.

1. – Harrel’s c-index, with correction for dependent censoring

Harrell’s c-index are biased in case of dependent censoring. Therefore, we use a C-index with correction for dependent censoring proposed by (1). These allow for unbiased estimation of the expected Brier score and the concordance index when a consistent estimator of the conditional censoring distribution is available and the conditional probability of remaining uncensored is positive at the evaluation time.

C-indices were calculated as

$${\hat{\boldsymbol{C}}}_{\boldsymbol{ipcw}}\left( \boldsymbol{t} \right)\boldsymbol{=}\frac{\sum_{\boldsymbol{i=1}}^{\boldsymbol{m}} \sum_{\boldsymbol{j=1}}^{\boldsymbol{m}} \tilde{\boldsymbol{N}_{\boldsymbol{ij}}}\boldsymbol{Q}_{\boldsymbol{n}}^{\boldsymbol{i,j}}\left( \boldsymbol{t} \right)\tilde{\boldsymbol{Y}_{\boldsymbol{i}}}\left( \boldsymbol{t} \right)\boldsymbol{\Delta}_{\boldsymbol{i}}^{\boldsymbol{D}}\hat{\boldsymbol{W}_{\boldsymbol{ij}}^{\boldsymbol{-1}}}}{\sum_{\boldsymbol{i=1}}^{\boldsymbol{m}} \sum_{\boldsymbol{j=1}}^{\boldsymbol{m}} \tilde{\boldsymbol{N}_{\boldsymbol{ij}}\tilde{\boldsymbol{Y}_{\boldsymbol{i}}}}\left( \boldsymbol{t} \right)\boldsymbol{\Delta}_{\boldsymbol{i}}^{\boldsymbol{D}}\hat{\boldsymbol{W}_{\boldsymbol{ij}}^{\boldsymbol{-1}}}}$$

where $\tilde{N}_{ij}$ indicates that patient $i$ experiences an event before patient $j$, $Q_{n}^{i,j}\left( t \right)$ indicates that patient $i$ had a greater marker value than patient $j$ (and thus was at higher risk meaning pairs $i$ and $j$ were concordant), $\tilde{Y_{i}}\left( t \right)$ indicates that patient $i$ experienced an event before truncation time $t$, and $\Delta_{i}^{D}$ indicates that patient $i$ died on the waiting list. $\hat{W_{ij}^{-1}}$ is the estimated weight of observing patient pair $i, j$ at time $i$ (see (16)). To correct for dependent censoring, these weights are set to

$$\hat{W_{ij}^{-1}}=\hat{G}\left( X_{i} | Z_{i}\left( s \right),s<X_{i} \right)\hat{G}\left( X_{i} | Z_{j}\left( s \right),s<X_{i} \right),$$

i.e. the product of the conditional probabilities of remaining uncensored for patient $i$ and $j$ at the event time of unit $i$.
